# Artificial Intelligence‐Assisted Cephalometric Analysis: A Comparative Study Between WebCeph and NemoCeph

**DOI:** 10.1155/ijod/3319286

**Published:** 2026-08-10

**Authors:** Ana Catarina Oliveira, Sofia Sousa-Santos, Joana Mendes, Selma Pascoal, Aline Gonçalves, José Manuel Mendes, Primavera Sousa-Santos

**Affiliations:** ^1^ UNIPRO – Oral Pathology and Rehabilitation Research Unit, University Institute of Health Sciences (IUCS), CESPU, Gandra 4585-116, Portugal, cespu.pt

**Keywords:** angular measurements, artificial intelligence, cephalometry, linear measurements, orthodontics, WebCeph

## Abstract

The incorporation of artificial intelligence (AI) into orthodontics has facilitated automated cephalometric assessments, offering greater efficiency and minimizing human‐related inaccuracies. Nevertheless, the precision of AI‐driven systems, when compared with conventional digital analysis software, requires thorough validation. To evaluate the accuracy and consistency of AI‐assisted cephalometric analysis using WebCeph in relation to manual tracings performed using NemoCeph, which is regarded as the reference standard. Cephalometric radiographs were obtained from 50 adult patients. Linear and angular parameters were measured using NemoCeph and WebCeph, and statistical comparisons were performed. Data normality was verified using the Shapiro–Wilk test, while paired *t*‐tests identified significant discrepancies. For the linear variables, the most notable differences occurred in S‐Ar, AO‐BO, overbite, and A‐Na‐Pog, with WebCeph occasionally overestimating or underestimating the results relative to NemoCeph. Regarding angular parameters, significant variations were identified in the sella angle, gonial angle, upper gonial angle, lower gonial angle, SNA, SNB, ANB, lower facial height, mandibular plane to S‐Na angle, occlusal plane to S‐Na angle, upper incisor to Na‐A angle, lower incisor to Na‐B angle, and lower incisor inclination. Differences were also statistically significant in determining the facial typology. WebCeph yields dependable outcomes for linear dental evaluations but demonstrates marked discrepancies in angular and vertical skeletal dimensions compared with NemoCeph. These findings underscore the importance of cautious clinical interpretation and suggest that AI‐based cephalometric tools should augment, rather than replace, expert orthodontic assessments.

## 1. Introduction

In recent years, the use of technology in dentistry has significantly increased, contributing to cost reduction, time optimization, decreased dependence on specific human skills, and the prevention of medical errors [1, 2]. Artificial intelligence (AI) refers to technological systems that imitate certain human cognitive functions such as reasoning, interpretation, and creative thinking [3]. AI has led to remarkable progress in various areas of medicine, allowing for a more precise evaluation of disease predisposition, more accurate diagnoses, and selection of the most appropriate treatments for each patient [4, 5].

In the field of dentistry, AI has been applied to several tasks, including tooth numbering, analysis of the relationship between third molars and the mandibular canal, implant planning, diagnosis of periapical pathologies, and detection of carious lesions [2, 4]. Moreover, AI has been increasingly implemented in orthodontics, particularly in orthodontic treatment planning and cephalometric analysis [3, 6].

Orthodontic treatment requires a comprehensive diagnostic approach involving the collection of various clinical records such as intraoral and extraoral photographs, radiographs, and study models [7, 8]. Among these, two‐dimensional (2D) lateral radiographs are of particular importance as they form the basis for cephalometric analysis [8]. Since its introduction in 1931 by Broadbent and Hofrath, cephalometric analysis has played a fundamental role in diagnosis, treatment planning, and evaluation of treatment outcomes [2]. Over time, cephalometric analysis has evolved with the incorporation of advanced technologies aimed at improving the accuracy and effectiveness of measurements [4, 9].

This technique relies on linear and angular measurements obtained from 2D lateral radiographs of the skull to create a cephalogram for each patient. Cephalometric landmarks are traditionally located on the skeletal structures, teeth, and soft tissues. The distances and angles between these points, together with reference planes and axes, allow for the classification of patients according to their skeletal, dental, and facial profile characteristics [10].

Traditionally, cephalometric tracing was performed manually using acetate paper and a ruler or protractor, which is a time‐consuming and operator‐dependent process prone to human error. The accuracy of this procedure could be influenced by radiographic quality and operator factors, such as fatigue and experience [11]. Imprecise landmark identification may compromise the reliability of cephalometric measurements and consequently, orthodontic diagnosis and treatment planning [10].

With the advent of digital technology, computer‐assisted cephalometry was introduced to overcome these limitations, providing greater consistency, reduced analysis time, and more accurate and reproducible measurements [12]. Initially, digital tracing software such as NemoCeph Version 20.10.0 (NEMOTEC, Madrid, Spain) enabled manual identification of landmarks in digital images, supported by tools for customized measurement and analysis [4, 13]. More recently, AI‐based platforms like WebCeph Version 2.0.0 (AssembleCircle Corp., Gyeonggi‐do, Republic of Korea) have been developed, offering fully automated landmark detection, rapid measurement generation, and visualization of treatment simulations [14].

The integration of AI into lateral cephalometry represents a promising opportunity to reduce human error, automate analysis, enhance diagnostic accuracy, and minimize measurement bias, particularly when assessing orthodontic treatment outcomes. Despite these advantages, the performance of automated systems can be affected by factors, such as the stage of dental development, congenital anomalies, and image quality [15].

Therefore, it is essential to evaluate the accuracy of AI‐generated measurements in comparison with those of traditional manual methods. The present study aimed to compare linear cephalometric measurements obtained automatically using WebCeph with those acquired manually using NemoCeph to assess the precision and reliability of automated analysis.

This study aimed to evaluate the accuracy of AI applications in orthodontics, specifically in cephalometric analysis. We hypothesized that no differences were to be found between NemoCeph and WebCeph. For this purpose, cephalometrics from 50 patients were analyzed, comparing the linear and angular values of different types of analysis obtained using NemoCeph, a conventional software program, and WebCeph, an AI‐based platform. For this study, NemoCeph was used as the conventional digital reference.

## 2. Materials and Methods

### 2.1. Study Design and Ethical Approval

This retrospective diagnostic study was conducted to compare the accuracy of cephalometric measurements obtained using a conventional digital software program (NemoCeph) and an AI‐based platform (WebCeph). The study was approved by the Ethics Committee of the University Institute of Health Sciences (01/CE‐IUCS/2025) on April 10, 2025. All participants provided verbal and written informed consent.

### 2.2. Participants and Data Collection

Cephalometric radiographs from 50 randomized patients were selected as auxiliary diagnostic tools prior to the orthodontic treatment. Radiographs were acquired between January 2022 and December 2024. All patients were treated by an orthodontic specialist in Portugal.

Patients included in the study were required to be 18 years old or older, present with complete permanent dentition (excluding third molars), and show Class I, II, or III malocclusions. Cephalometric radiographs had to be of high quality and free from artifacts that could interfere with the identification of anatomical landmarks. All participants voluntarily agreed to participate in the study by signing an informed consent form, and none had undergone previous orthodontic treatment.

The exclusion criteria comprised the absence of first molars or central incisors, extensive crown‐bridge restorations, and the presence of dental implants in the region of the incisors or first molars. Patients were also excluded if they had any type of maxillofacial or orthognathic surgery or presented with craniofacial anomalies or congenital dentofacial syndromes.

### 2.3. Software Used in This Study

WebCeph Version 2.0.0 (AssembleCircle Corp., Gyeonggi‐do, Republic of Korea) is an AI‐based digital platform that enables automated cephalometric analysis by recognizing anatomical landmarks and generating measurements within seconds. It supports orthodontic and surgical planning, offers treatment simulations, and allows free tracing, making it a cost‐effective alternative to conventional software.

The workflow within the platform consists of several sequential steps. First, a patient profile is created by entering demographic and clinical information. Subsequently, the patient’s lateral cephalometric radiograph is uploaded to the system. Once the image is available, the software performs automatic landmark digitalization through its AI‐based tracing system, identifying cephalometric reference points without the need for manual plotting. After image processing, the user can access different cephalometric analyses, including Steiner, McNamara, Ricketts, Jarabak, and Tweed analyses, among others. The software automatically calculates the corresponding angular and linear measurements and generates a complete cephalometric report that can be reviewed, downloaded, and stored digitally. WebCeph does provide users with the option to manually adjust or correct the automatically detected landmarks. However, in the present study, no manual corrections were applied, as our objective was to evaluate and compare a fully AI‐based cephalometric analysis system with conventional manual tracing.

NemoCeph Version 20.10.0 (NEMOTEC, Madrid, Spain), the conventional digital reference, is a widely used cephalometric software that provides precise manual tracings for orthodontic diagnosis and treatment planning. It allows the superimposition of tracings for progress evaluation and ensures reliable results; however, its main limitation is the cost of the software.

Both methods were applied independently to the same set of images.

### 2.4. Cephalometric Landmarks Used for NemoCeph Tracing

Table 1 summarizes the hard‐ and soft‐tissue cephalometric landmarks used for tracing in NemoCeph.

**Table 1:** Cephalometric landmarks of hard and soft tissues used in this study for tracing with NemoCeph.

Landmark	Definition
Nasion (Na)	Most anterior point of the frontonasal suture
Sella (S)	Center of the sella turcica (pituitary fossa)
Mentonian (Me)	Lowest point on the mandibular symphysis
Orbitale (O)	Lowest point on the inferior margin of the orbit
Porion (Po)	Highest point of the external auditory meatus
Pterygomaxillary fissure (Pt)	Point at the base of the pterygomaxillary fissure
Posterior nasal spine (PNS)	Tip of the posterior nasal spine
Anterior nasal spine (ANS)	Tip of the anterior nasal spine
Subspinale (A)	Deepest point on the curvature of the anterior maxilla
Basion (Ba)	Most inferior‐posterior point on the anterior border of the foramen magnum
Antegonial notch (Ag)	Concavity on the lower border of the mandible anterior to the gonial angle
Gonion (Go)	Most posterior‐inferior point at the mandibular angle
Condylion (Co)	Most superior point on the mandibular condyle
Articulare (Ar)	Intersection between the posterior border of the ramus and the cranial base
Upper right molar distal (A6D)	Distal point of the maxillary first molar
Upper right molar mesial (A6M)	Mesial point of the maxillary first molar
Lower right molar distal (B6D)	Distal point of the mandibular first molar
Lower right molar mesial (B6M)	Mesial point of the mandibular first molar
Upper incisor (A1)	Incisal tip of the most prominent upper incisor
Lower incisor (B1)	Incisal tip of the most prominent lower incisor
Upper incisor root apex (A2)	Root apex of the upper incisor
Lower incisor root apex (B2)	Root apex of the lower incisor
Occlusal plane posterior	Posterior intersection of occlusal plane, usually between distal molars
Occlusal plane anterior	Anterior intersection of occlusal plane, usually at incisal edges
Pogonion (Pg)	Most anterior point of the bony chin
Suprapogonion (Pm)	Depression between Point B and pogonion
Supramentale (B)	Deepest point on the anterior contour of the mandibular symphysis
Soft tissue menton (Me′)	Soft tissue counterpart of menton
Soft tissue gnathion (Gn′)	Midpoint between soft tissue pogonion and menton
Soft tissue pogonion (Pg′)	Most anterior point on the soft tissue chin
Inferior labial sulcus	Concavity between lower lip and chin
Lower lip vermilion	Most prominent point of the vermilion of the lower lip
Lower lip anterior (LL)	Most anterior point of the lower lip
Stomion inferior (Sti)	Median point at the lower lip line (vermilion border)
Lower lip internal	Inner surface of the lower lip, in contact with teeth
Upper lip internal	Inner surface of the upper lip, in contact with teeth
Stomion superior (Sts)	Median point at the upper lip line (vermilion border)
Upper lip anterior (UL)	Most anterior point of the upper lip
Superior labial sulcus	Concavity above the upper lip
Subnasale (Sn)	Junction between the columella and upper lip
Columella (Cm)	Most anterior point on the nasal columella
External nose	Most anterior point of the nasal tip (pronasale)
Soft tissue nasion (N′)	Soft tissue counterpart of nasion
Soft tissue glabella (G′)	Most prominent point on the soft tissue forehead in the midline

### 2.5. Determination of Facial Biotype

The facial biotype was determined for all 50 patients using Ricketts analysis based on the following parameters: facial axis, facial depth, mandibular plane angle, lower facial height, and mandibular arch. Since all participants were adults (≥18 years), craniofacial growth was considered complete. Standard values originally defined for 9‐year‐olds were adjusted according to Ricketts’ age correction factors (Table 2).

**Table 2:** Cephalometric parameters used for facial biotype classification according to Ricketts analysis.

Biotype	Standard 9 years	Adjustment	Years	Patient	Deviation	Sum	≥+1 severe brachyfacial
Facial axis	90° ± 3°	—	—	—	—	—	≥+0.5 severe brachyfacial
Facial depth	87° ± 3°	+ 0.3°/years	—	—	—	—	0 mesofacial
Mandibular plane angle	26° ± 4°	−0.3°/years	—	—	—	—	≥−0.5 soft dolichofacial
Lower facial height	47° ± 4°	—	—	—	—	—	≥−1 dolichofacial
Mandibular arc	26° ± 4°	+0.5°/years	—	—	—	—	≥−2 severe dolichofacial

### 2.6. Statistical Analysis of the Data

Statistical analysis was performed using SPSS software (Version 29.0; IBM Corp., 2023). Descriptive statistics included frequencies (*n*) and percentages (%) for categorical variables and means and standard deviations for continuous variables with normal distributions.

The normality of distributions was assessed using the Shapiro–Wilk test, which is appropriate for sample sizes equal to or lower than 50. Paired *t*‐tests were used to compare the measures and determine whether there were statistically significant differences between the measurements obtained using NemoCeph and Webceph. Sample size calculations were based on paired‐sample *t*‐tests, concerning 95% power, 5% statistical significance, and moderate effect size (Cohen’s *d* = 0.5).

The significance level considered for rejecting the null hypothesis was set at 5% (*p* < 0.05).

To assess the reliability of manual landmark identification in NemoCeph, intraobserver and interobserver reliability analyses were performed. A randomly selected subset of cephalometric radiographs was traced twice by the same observer, an orthodontic specialist, after an interval of at least 24 h and independently by a second observer. Intraclass correlation coefficients (ICCs) were calculated to assess the reproducibility of the measurements. The results revealed reliability ranging from good to excellent, confirming the consistency of the manual identification process for reference points in NemoCeph.

The ICC was calculated as a measure of agreement between the two measurement methods. Across all skeletal, vertical, dental, and soft‐tissue measurements, the ICCs revealed substantial variation in agreement between NemoCeph and WebCeph. Several skeletal linear variables demonstrated very low concordance, with ICC values close to zero, as seen for the anterior cranial base (ICC = 0.03, 95% CI −0.25 to 0.30), posterior cranial base (ICC = 0.05, 95% CI −0.23 to 0.32), mandibular body length (ICC = 0.10, 95% CI −0.18 to 0.37), and mandibular length and maxillary length, both showing ICCs below 0.10. In contrast, angular skeletal indicators generally performed better, with high agreement for SNA (ICC = 0.73), SNB (ICC = 0.83), ANB (ICC = 0.91), the mandibular plane angle to SN (ICC = 0.93), and the mandibular plane angle (ICC = 0.82). Moderate agreement was observed for angles such as the saddle angle (ICC = 0.41), the upper and lower gonial angles (ICC = 0.57 and 0.63, respectively), while the articular angle showed virtually no reliability (ICC = −0.04). Within the vertical dimension, the anterior facial height exhibited no correlation (ICC = 0.00), and the posterior facial height showed only weak agreement (ICC = 0.22). In contrast, the lower facial height demonstrated high reliability (ICC = 0.83), and vertical angular measures such as FMA, FMIA, and the palatal plane angle showed moderate ICCs ranging from 0.58 to 0.61. Dental measures overall yielded stronger agreement, with AO–BO (ICC = 0.81), overjet (ICC = 0.69), and IMPA (ICC = 0.77) demonstrating moderate to high concordance, while several upper and lower incisor angulations—including U1 to NA (°), L1 to NB (°), and L1 to A–Pog (°)—ranged from moderate to strong (ICC values between 0.53 and 0.76). The occlusal plane to SN presented a high ICC of 0.85, and the cant of the occlusal plane also performed strongly (ICC = 0.83). However, the interincisal angle showed only modest agreement (ICC = 0.39), and the upper molar position displayed weak concordance (ICC = 0.14). Protrusion measures varied, with lower incisor protrusion showing excellent reliability (ICC = 0.88), whereas several others fell into low‐ or moderate categories. For soft‐tissue analysis, facial convexity demonstrated excellent agreement (ICC = 0.89), contrasting sharply with lip protrusion, which showed extremely poor reliability (ICC = 0.04). Overall, the ICC values indicated high consistency between the two software systems for angular skeletal measurements, several vertical and dental angular parameters, and key soft‐tissue angles, while linear skeletal variables and selected soft‐tissue and dental measures revealed weak‐to‐negligible agreement.

## 3. Results

The study included a total of 50 adult participants, with a mean age of 31 years. Among them, 16 were males, with a mean age of 30 years, and 34 were females, with a mean age of 29 years. These demographic data demonstrate that a balanced adult sample was suitable for comparative analysis. These results are presented in Table 3.

**Table 3:** Characteristics of the clinical study sample (age and gender distribution).

Characteristics	*n*	Mean age (years)
Age (years)	50	31
Male	16	30
Female	34	29

All cephalometric variables and their comparative statistical outcomes are summarized in Table 4, and the main findings are described as follows.

**Table 4:** Comparisons between NemoCeph and WebCeph.

Measurement type	Variable	NemoCeph (mean ± SD) (mm or °)	WebCeph (mean ± SD) (mm or °)	(NemoCeph – WebCeph) (mm or °)	*p*‐Value	ICC (95% CI)
Skeletal	Anterior cranial base (mm)	67.54 (18.33)	69.94 (1.06)	−2.40 (18.09)	0.353	0.03 [−0.25, 0.30]
	Posterior cranial base (mm)	30.18 (9.52)	35.99 (7.63)	−5.81 (11.89)	0.001 ^∗∗^	0.05 [−0.23, 0.32]
	Mandibular body length (mm)	70.98 (19.42)	73.58 (4.76)	−2.60 (18.97)	0.337	0.10 [−0.18, 0.37]
	Mandibular ramus height (mm)	47.26 (15.50)	49.37 (6.12)	−2.12 (13.58)	0.276	0.34 [0.07, 0.56]
	SNA (°)	80.36 (3.96)	82.19 (3.65)	−1.83 (2.80)	<0.001 ^∗∗∗^	0.73 [0.57, 0.84]
	SNB (°)	75.92 (4.27)	77.13 (4.09)	−1.21 (2.45)	0.001 ^∗∗^	0.83 [0.72, 0.90]
	ANB (°)	4.46 (2.39)	5.06 (2.43)	−0.60 (1.04)	<0.001 ^∗∗∗^	0.91 [0.84, 0.95]
	Mandibular length (mm)	112.53 (31.24)	115.32 (5.91)	−2.79 (30.62)	0.523	0.07 [−0.21, 0.34]
	Maxillary length (mm)	85.31 (24.15)	87.00 (3.52)	−1.68 (23.46)	0.614	0.08 [−0.20, 0.34]
	Mandibular plane angle to SN (°)	35.20 (7.49)	31.50 (7.34)	3.70 (2.71)	<0.001 ^∗∗∗^	0.93 [0.89, 0.96]
	Mandibular plane angle (°)	24.54 (7.43)	22.73 (8.21)	1.30 (2.49)	0.009 ^∗∗^	0.82 [0.70, 0.89]
	Saddle angle (°)	122.76 (6.89)	125.05 (5.31)	−2.29 (6.68)	0.019 ^∗^	0.41 [0.15, 0.62]
	Articular angle (°)	134.32 (79.55)	148.54 (5.43)	−14.22 (81.27)	0.222	−0.04 [−0.31, 0.24]
	Gonial angle (°)	117.08 (10.05)	119.42 (7.86)	−2.34 (6.35)	0.012 ^∗^	0.75 [0.60, 0.85]
	Upper gonial angle (°)	43.24 (6.89)	46.73 (3.99)	−3.49 (5.24)	<0.001 ^∗∗∗^	0.57 [0.34, 0.73]
	Lower gonial angle (°)	73.70 (6.20)	71.69 (8.33)	2.01 (6.31)	0.029 ^∗^	0.63 [0.43, 0.77]
	Facial depth angle (°)	87.96 (3.81)	86.01 (12.92)	0.23 (1.83)	0.255	0.21 [−0.07, 0.46]
	Facial axis (°)	87.84 (4.89)	85.73 (13.32)	0.39 (2.12)	0.245	0.20 [−0.08, 0.45]
	Mandibular arc (°)	38.06 (7.21)	36.81 (6.16)	1.25 (5.19)	0.095	0.70 [0.53, 0.82]

Vertical	Anterior facial height (mm)	114.79 (31.97)	121.29 (7.35)	−6.50 (32.80)	0.167	0.00 [−0.28, 0.28]
	Posterior facial height (mm)	75.32 (22.33)	81.16 (8.16)	−5.84 (21.01)	0.055	0.22 [−0.06, 0.47]
	Lower facial height (mm)	44.84 (5.42)	46.33 (5.00)	−1.49 (3.00)	0.001 ^∗∗^	0.83 [0.73, 0.90]
	FMA (°)	26.18 (6.64)	24.97 (10.23)	1.21 (7.77)	0.278	0.59 [0.38, 0.75]
	FMIA (°)	58.38 (9.00)	59.06 (11.64)	−0.68 (9.21)	0.606	0.61 [0.40, 0.76]
	Palatal plane angle (°)	−1.58 (3.58)	−0.99 (2.03)	−0.62 (2.66)	0.129	0.58 [0.36, 0.74]

Dental	AO–BO (mm)	1.40 (3.55)	2.60 (3.57)	−1.20 (2.22)	<0.001 ^∗∗∗^	0.81 [0.68, 0.88]
	Overjet (mm)	4.20 (2.28)	4.26 (1.64)	−0.06 (1.58)	0.792	0.69 [0.51, 0.81]
	Overbite (mm)	3.60 (3.04)	2.46 (2.11)	1.14 (2.54)	0.003 ^∗∗^	0.53 [0.30, 0.70]
	U1 to NA (mm)	3.44 (2.97)	3.16 (1.74)	0.29 (2.97)	0.501	0.26 [−0.02, 0.50]
	U1 to NB (mm)	5.91 (4.17)	5.85 (2.52)	0.06 (3.02)	0.887	0.62 [0.41, 0.76]
	U1 to NA (°)	21.14 (6.55)	18.01 (6.42)	3.13 (4.47)	<0.001 ^∗∗∗^	0.76 [0.62, 0.86]
	L1 to NB (°)	28.39 (8.30)	26.25 (6.74)	2.14 (5.25)	0.006 ^∗∗^	0.76 [0.61, 0.86]
	L1 to A–Pog (°)	25.56 (6.11)	22.17 (5.70)	2.74 (4.47)	<0.001 ^∗∗∗^	0.53 [0.30, 0.70]
	IMPA (°)	96.34 (7.99)	96.16 (7.52)	0.18 (5.29)	0.816	0.77 [0.62, 0.86]
	Occlusal plane to SN (°)	18.42 (5.75)	16.42 (5.18)	2.00 (2.99)	<0.001 ^∗∗∗^	0.85 [0.75, 0.91]
	Cant of occlusal plane (°)	7.26 (5.01)	6.84 (4.91)	0.42 (2.86)	0.300	0.83 [0.73, 0.90]
	Interincisal angle (°)	125.76 (10.96)	128.57 (21.45)	−5.05 (5.92)	0.297	0.39 [0.13, 0.60]
	Upper molar position (mm)	17.95 (7.27)	17.90 (4.59)	0.05 (8.00)	0.965	0.14 [−0.15, 0.40]
	Lower incisor protrusion (mm)	2.16 (2.53)	1.91 (2.45)	0.26 (1.22)	0.142	0.88 [0.80, 0.93]
	Upper incisor protrusion (mm)	6.26 (3.33)	6.06 (2.76)	0.20 (2.10)	0.501	0.76 [0.62, 0.86]

Soft tissue	Facial convexity (°)	3.43 (2.75)	4.17 (3.01)	−0.74 (1.39)	<0.001 ^∗∗∗^	0.89 [0.81, 0.93]
	Lip protrusion (mm)	1.26 (11.92)	−1.70 (2.65)	2.95 (11.95)	0.087	0.04 [−0.24, 0.31]

^∗^ *p* < 0.05.

^∗∗^ *p* < 0.01.

^∗∗∗^ *p* < 0.001.

No significant differences were found in skeletal linear measurements of the anterior cranial base (*p* = 0.353), mandibular body length (*p* = 0.337), or mandibular ramus height (*p* = 0.276). However, the posterior cranial base (*p* = 0.001) was significantly higher in WebCeph, indicating a systematic difference between the software programs.

Among the angular skeletal parameters, the saddle angle (*p* = 0.019), gonial angle (*p* = 0.012), and upper gonial angle (*p* < 0.001) were significantly lower in NemoCeph, while the lower gonial angle (*p* = 0.029) was higher. No significant difference was observed in the articular angle (*p* = 0.222). The mandibular plane angle (*p* = 0.009) and the mandibular plane to SN (*p* < 0.001) were significantly higher in NemoCeph, and the mandibular arc presented a marginally higher value (*p* = 0.095).

Vertical parameters showed no significant differences in anterior facial height (*p* = 0.167) or posterior facial height (*p* = 0.055), although the latter tended to be higher with WebCeph. Lower facial height (*p* = 0.001) was significantly lower in NemoCeph. No significant differences were observed in the angular vertical parameters, including FMA (*p* = 0.278), FMIA (*p* = 0.606), facial axis (*p* = 0.245), facial depth angle (*p* = 0.255), and palatal plane angle (*p* = 0.129).

For dental measurements, linear variables, such as U1 to NA (*p* = 0.501), U1 to NB (*p* = 0.887), overjet (*p* = 0.792), upper molar position (*p* = 0.965), lower incisor protrusion (*p* = 0.142), and upper incisor protrusion (*p* = 0.501) showed no significant differences. Lip protrusion showed a marginal difference (*p* = 0.087), suggesting a slight tendency for WebCeph to yield lower values. AO–BO (*p* < 0.001) was significantly higher in WebCeph while overbite (*p* = 0.003) was significantly lower. The interincisal angle (*p* = 0.297) did not differ significantly.

Angular dental parameters showed significant differences for the upper incisor to Na‐A plane (*p* < 0.001), the lower incisor to Na‐B (*p* = 0.006), and the upper incisor to A–Pog (*p* < 0.001), all of which were higher in NemoCeph. No significant differences were found in the IMPA (*p* = 0.816).

Maxillary length (*p* = 0.614) and mandibular length (*p* = 0.523) did not differ significantly between the two software programs. Similarly, the cant of the occlusal plane (*p* = 0.300) showed no significant difference.

Regarding maxillomandibular relationships, the SNA (*p* < 0.001), SNB (*p* = 0.001), and ANB (*p* < 0.001) angles were significantly lower in NemoCeph, while the occlusal plane to S‐Na plane (*p* < 0.001) and mandibular plane to S‐Na plane (*p* < 0.001) angles were significantly higher. There was no significant difference in the cant of the occlusal plane (*p* = 0.300). Facial convexity (*p*  < 0.001) was significantly higher in WebCeph than in NemoCeph.

**Figure 1:**
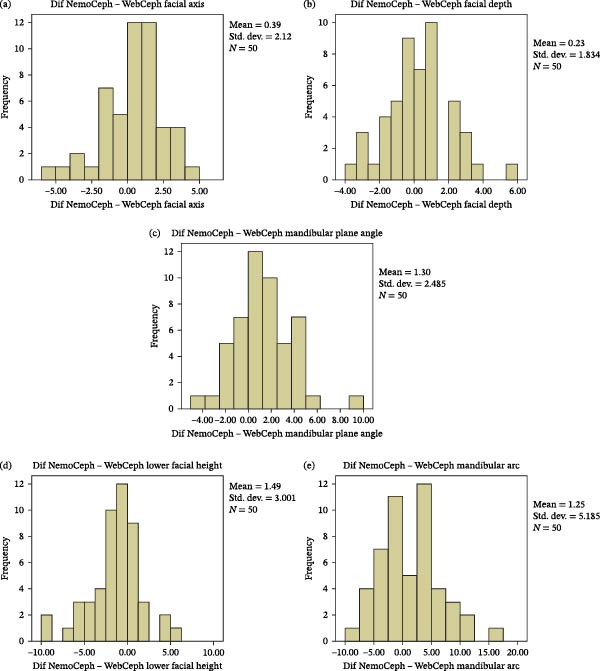
Histograms illustrating the distribution of differences in angular measurements between NemoCeph and WebCeph (NemoCeph – WebCeph) for five cephalometric parameters from the Ricketts analysis used for facial biotype determination: (a) facial axis, (b) facial depth, (c) mandibular plane angle, (d) lower facial height, and (e) mandibular arc. Each plot displays the mean, standard deviation (SD), and sample size (*N* = 50), highlighting the variability and direction of discrepancies between the two software tools for each parameter.

The facial biotype was determined based on measurements obtained from WebCeph and NemoCeph. The observed differences were the result of discrepancies between the two software programs in the measurements required to classify the biotypes. Figure 1 illustrates the differences between NemoCeph and WebCeph for the five variables used to establish facial biotypes.

## 4. Discussion

Cephalometric analysis provides essential information for orthodontic diagnosis, treatment planning, and outcome assessment. Accurate measurements are crucial for developing appropriate treatment strategies and minimizing the risk of incorrect interventions. With the increasing incorporation of AI into orthodontic tools, it is vital to validate AI‐based software against traditional methods to ensure reliability and patient safety [2, 3].

This study evaluated the differences between two digital cephalometric software programs, NemoCeph and WebCeph, in obtaining classical cephalometric measurements, with a specific focus on the Jarabak, McNamara, Steiner, Ricketts, and Tweed analyses [16–20]. The comparison between the two measurement methods, taking NemoCeph as the conventional digital reference, aimed to determine whether significant differences exist between the two approaches in capturing linear and angular cephalometric data.

Despite the increasing integration of AI into orthodontic diagnostic tools, it remains difficult to find studies that directly compare WebCeph with NemoCeph, particularly using adult clinical samples. The existing literature often contrasts WebCeph with other conventional software programs, which, despite similar diagnostic aims, are not methodologically identical to NemoCeph. This gap highlights the scientific relevance of the present study and frames its findings within a context that still lacks standardized validation between these two specific tools [15].

The results demonstrate that while some variables display minor and statistically nonsignificant differences, others show substantial discrepancies. The accuracy of AI‐based cephalometric systems may be influenced by factors such as radiographic quality, landmark visibility, craniofacial morphology, and the anatomical complexity of posterior and vertical skeletal structures, which may contribute to variability in automated landmark identification and measurements. These findings suggest that the two methods may not be entirely equivalent in measuring certain craniofacial structures, thereby supporting the stated null hypothesis [2].

Significant discrepancies were observed in the angular variables: the saddle, gonial, and upper gonial angles were higher with WebCeph, whereas the lower gonial angle was higher with NemoCeph; no relevant differences were found in the articular angle. Regarding the linear measurements of the anterior cranial base, mandibular length, ramus height, anterior facial height, and posterior facial height, no significant differences were observed, except for the posterior cranial base (*p* = 0.001), which was higher in WebCeph. These findings align with previous reports that AI‐based software tends to overestimate angles and linear dimensions in complex craniofacial structures [8, 21].

No statistically significant differences were observed in the angular measurements of FMIA, FMA, and IMPA (*p* > 0.2), indicating good agreement between the two software programs, in line with Prince et al. [14]. However, the linear variable AO‐BO presented statistically and clinically significant differences (*p* < 0.001), consistent with the findings of Mercier et al. [8]. This is noteworthy because AO‐BO is particularly sensitive to the accurate identification of points A and B, which may vary depending on the software used.

In addition, we observed that the differences in the SNA, SNB, and ANB angles were significantly higher with WebCeph (*p* < 0.001), whereas the mandibular plane to S‐Na and occlusal plane to S‐Na angles were lower. For linear dental measurements, no significant discrepancies were found between the upper incisor to Na‐A plane or the upper incisor to Na‐B plane. These findings agree with those of Mercier et al. [8], Baig et al. [15], and Kazimierczak et al. [22], who also reported inconsistencies in these variables, highlighting the limitations of AI‐based systems in the accurate localization of skeletal and dental landmarks such as the menton, gonion, orbitale, orion, and nasion.

Other discrepancies were associated with facial type assessment. NemoCeph reported higher values for lower facial height, mandibular plane angle, and lower incisors to A‐Pog (*p* < 0.001). In contrast, facial depth, facial axis, interincisal angle, palatal plane angle, and mandibular arc showed no significant differences. This pattern is consistent with that of Zaheer et al. [23], who reported consistency in interincisal measures but higher SNA values in WebCeph. By contrast, El‐Dawlatly et al. [24] found significant differences in nearly all variables when comparing WebCeph with OnyxCeph, reinforcing the limitations of AI‐based systems in vertical and posterior cephalometric measurements. Clinically, these discrepancies impacted the classification of facial type; WebCeph identified 24 patients as severe brachyfacial, compared to 28 using NemoCeph.

Comparisons with the literature reinforce these observations. Chuchra et al. [12] found no significant differences between NemoCeph and WebCeph for linear measures, including anterior facial height and incisor positions, supporting the present results. For example, Baig et al. [15], Prince et al. [14], and El‐Dawlatly et al. [24] reported a strong concordance for linear dental variables. Çoban et al. [21], however, observed significant differences for ANS‐Me, upper incisor to Na‐A, and lower incisor to Na‐B when comparing WebCeph with Dolphin Imaging, highlighting that divergence patterns may depend on the reference software and study population.

Although the sample presented an unequal sex distribution, the cephalometric radiographs were randomly selected independently of sex. Since the objective of this study was to compare AI‐assisted and manual cephalometric measurements based on the same anatomical landmarks, sex was not expected to directly influence the methodological comparison.

Importantly, statistically significant differences do not necessarily correspond to clinically meaningful effects, highlighting the importance of clinician oversight when interpreting AI‐based cephalometric measurements.

The clinical relevance of the present findings should therefore be considered beyond statistical significance alone. Although several variables demonstrated significant differences between the two software programs, many of these discrepancies may have a limited impact on orthodontic diagnosis or treatment planning in routine clinical situations.

Nevertheless, measurements related to sagittal skeletal relationships, vertical facial proportions, and facial type classification play a critical role in decisions concerning growth pattern evaluation, extraction protocols, orthognathic surgery planning, and biomechanical strategies. Consequently, even small deviations in landmark identification or angular interpretation by AI‐based systems may compromise diagnostic accuracy, particularly in borderline or complex clinical cases.

However, this study has some limitations. The sample included only adult patients; therefore, the results may not be generalizable to a growing population. Furthermore, only one AI‐based software package was used, limiting the extrapolation of the results to other platforms. Thus, further retrospective and clinical studies based on other AI platforms are essential for the advancement of this field.

## 5. Conclusion

In conclusion, while NemoCeph and WebCeph exhibit an overall agreement in linear dental measurements, discrepancies in angular and vertical skeletal parameters underscore the importance of careful clinical interpretation, particularly for facial biotype assessment and treatment planning. These findings highlight the need for further direct comparative research between these software platforms in adult populations to improve the reliability of digital cephalometric tools and support informed clinical decision‐making.

## Funding

No funding was received for this manuscript.

## Conflicts of Interest

The authors declare no conflicts of interest.

## Data Availability

The datasets generated and analyzed during the current study are not publicly available due to ethical and patient privacy restrictions but are available from the corresponding author upon reasonable request.
